# Self-Assessed Aspects of Health 3 Months after COVID-19 Hospitalization—A Swedish Cross-Sectional Study

**DOI:** 10.3390/ijerph19138020

**Published:** 2022-06-30

**Authors:** Alexandra C. Larsson, Marie Engwall, Annie Palstam, Hanna C. Persson

**Affiliations:** 1 Department of Clinical Neuroscience, Institute of Neuroscience and Physiology, Sahlgrenska Academy, University of Gothenburg, 413 45 Gothenburg, Sweden; marie.engwall@hv.se (M.E.); annie.palstam@gu.se (A.P.); hanna.persson@neuro.gu.se (H.C.P.); 2 Department of Occupational Therapy and Physical Therapy, Sahlgrenska University Hospital, 413 46 Gothenburg, Sweden; 3 Department of Health Sciences, University West, 461 32 Trollhattan, Sweden; 4 Department NeuroScience, Sahlgrenska University Hospital, 413 46 Gothenburg, Sweden; 5 School of Health and Welfare, Dalarna University, 791 31 Falun, Sweden

**Keywords:** COVID-19, rehabilitation, recovery of function, self-assessment, public health

## Abstract

It is not yet fully understood how the patients self-assess their overall health in the early recovery after COVID-19 and if certain patient groups are more prominent in perceived long-time effects of COVID-19. The aim of this study was to describe self-assessed aspects of health in body function, activity and participation 3 months after hospitalization due to COVID-19 and identify difference between groups depending in age, sex and level of hospital care. This cross-sectional study consists of self-assessed aspects of health and recovery in 168 participants (mean age 64 years old, 69% men) previously hospitalized patients due to COVID-19. We have previously published data, from hospital discharge, on this cohort were predominantly the older patients and previous ICU-treated participants were affected. In this study there were differences in between groups. Of the study population 72% perceived fatigue, 64% respiratory difficulties, 37% perceived symptoms of anxiety. Three-months after COVID-19 this cohort was overall still affected. The recovery process is multifaced and the cohort heterogeneous, hence the rehabilitation needs to be highly individualized, and the follow-up of this patient group is of importance regardless of age, sex and previous level of hospital care.

## 1. Introduction

Consequences of severe COVID-19 infection may require hospitalization [1] and result in impaired physical and cognitive function, as well as limitations in activities of daily living [2,3,4]. These impairments have been shown to be most prominent in elderly patients and patients who have required treatment in intensive care units (ICUs) [2]. Although COVID-19 patients benefit from in-hospital rehabilitation [4], most patients do not qualify for this. Studies have shown promising results in recovery from COVID-19 [5,6], whereas others point out that some groups may still suffer from persisting respiratory impairments [7] and have low participation in society [8]. In-hospital care for COVID-19 has also been suggested to be a predictor of long sick leave [9]. Continued follow-up after discharge from in-patient care is important in order to evaluate changes in functioning and recovery and the need for rehabilitation [3].

To understand the consequences of COVID-19, follow-up studies have been urged to include aspects of function, activity, and participation captured in the biopsychosocial model from the International Classification of Disability and Health (ICF) [10]. Patient-reported outcome measures (PROMs) could be used for individual evaluations post COVID-19 in order to incorporate different ICF domains. PROMs are used to capture patients’ perceptions of their situation [11] and are recommended for patients with COVID-19 [12]. As the population of people recovering from COVID-19 grows, it is necessary to understand their problems and how they are affected [13]. Various impairments in body functions, such as respiratory impairments and sleeping disorders, may be persistent symptoms after COVID-19 [7,14]. Furthermore, patients in recovery after COVID-19 may suffer from reduced quality of life [12,14,15,16]. Though it seems to be clear that various difficulties may occur during the recovery process after COVID-19, how patients perceive their overall health in the early recovery period, whether any domains in the ICF model [17] are more affected, or whether certain patient groups perceive more prominent long-term effects of COVID-19 is not yet fully understood [18].

The aim of the present study was to describe self-assessed aspects of health in body function, activity, and participation 3 months after hospitalization due to COVID-19 and identify differences between groups depending on age, sex, and level of hospital care.

## 2. Materials and Methods

### 2.1. Patients

This cross-sectional study included patients enrolled in the longitudinal project “Life in the time of Covid study in Gothenburg” (GOT-LOCO) [2]. The project includes 211 patients treated for COVID-19 in hospitals throughout the Västra Götalands region (VGR), a catchment area of 1.67 million, in Sweden. Patients were recruited with an intent of consecutive recruitment from five hospitals with a total of nine units within the VGR. The inclusion was carried out between 9 July 2020 and 23 February 2021. Inclusion criteria were age ≥18 years and hospitalization for COVID-19 within the VGR but non-contagious at inclusion with an expected hospital care period >5 days after possible ICU treatment. Patients had to have previously lived independently in the community. If the patient was not able to give informed consent or had severe illness with expected high 1-year mortality they were excluded from the study. Non-Swedish residents were also excluded. 

The study was approved by the Swedish Ethical Review Authority (Drn: 2020-0346, 2020-03922, 2020-00444) and complies with the declaration of Helsinki. The manuscript was constructed using the STROBE checklist. All patients signed informed consent prior to inclusion. 

### 2.2. Data Collection

**Figure 1:**
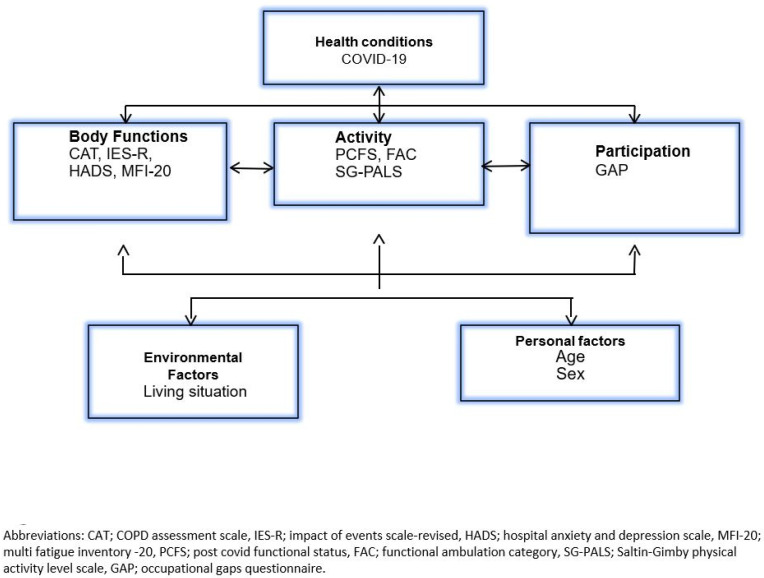
Patient evaluation according to the International Classification of functioning (ICF).

The patients from GOT-LOCO were contacted by phone approximately 3 months after discharge regarding participation in this study and to schedule a telephone interview. Prior to the interview, the PROMs were sent out and participants were later contacted by telephone at an agreed upon time for a structured telephone interview based on the PROMs. If a participant was not reached by phone, after at least three atempts, PROMS were sent out through the mail. Information regarding the patient’s hospital stay was retrieved from their medical records. PROMs were classified according to ICF domains [17] (Figure 1). 

### 2.3. Body Function

Respiratory function was self-assessed using the chronic obstructive pulmonary disease assessment test (CAT). The CAT is an 8-item ordinal scale with each item scored 0–5 points (total 0–40 points), with a higher score indicating severe respiratory difficulties [19]. In the present study, a cut-off ≥10 was used [20] to indicate respiratory difficulties. Self-assessed symptoms of psychological trauma specifically related to COVID-19 was captured using the Impact of Event Scale-Revised (IES-R) [21]. The scale ranges from 0–88 points, with a higher score indicating higher perceived self-rated traumatic stress [22]. The IES-R measures levels of intrusion, avoidance, and hyperarousal, and a cut-off ≥33 points indicates possible post-traumatic stress syndrome [22,23]. Symptoms related to anxiety and depression were self-assessed using the Hospital Anxiety and Depression scale (HAD) with a cutoff >7 points in each subscale, indicating the possible presence of anxiety and/or depression [24]. Self-perceived fatigue was evaluated using the Multidimensional Fatigue Inventory-20 (MFI-20). The MFI-20 includes five subscales: general fatigue, physical fatigue, reduced activity, reduced motivation, and mental fatigue (score 0–100 points) [23]. A higher score indicates more fatigue [23]. General fatigue and physical fatigue highly correlate [25] and, it has been argued that, it is difficult to distinguish between the two [23,25]. The total score for the subscale general fatigue was used in this analysis. A cut-off ≥11 was used for the subscale general fatigue, which is just above the normative values for men and women in the Swedish general population [26]. 

### 2.4. Activity and Participation 

Self-perceived physical status after COVID-19 was assessed using the Post Covid Functional Status scale (PCFS), 0, no functional limitations to 4, severe functional limitations [27]. Ambulation was assessed by the Functional Ambulation Category (FAC) [28], a 6-point scale (0, non-functional ambulator to 5, independent ambulator) that measures the level of physical support the individual needs while walking. Physical activity level since hospital discharge was assessed using the Saltin Gimby Physical Activity Level Scale (SG-PALS) (1, physically inactive to 4, regular hard physical training) [29]. Participation in daily life activities was self-assessed using the Occupational Gaps Questionnaire (OGQ) [30]. The patients reported experiencing want to do gaps (WTDG) in certain activities in four domains: instrumental activities of daily living (I-ADL), leisure activities, social activities, and work. 

### 2.5. Data Analysis

Descriptive statistical analysis was carried out for the entire population, as well as for sub-groups, using numbers and percentages, means and standard deviation (SD) as well as median and inter quartile range (IQR). The groups were defined by age (<65 years and ≥65 years), sex (man or woman), and level of hospital care (having received ICU-treatment or not). For group comparison, students T test for continuous data and the Mann Whitney U test for variables consisting of ordinal data were used. Data was processed in SPSS Statistics 28 (IBM Corporation, Armonk, NY, USA).

## 3. Results

**Figure 2:**
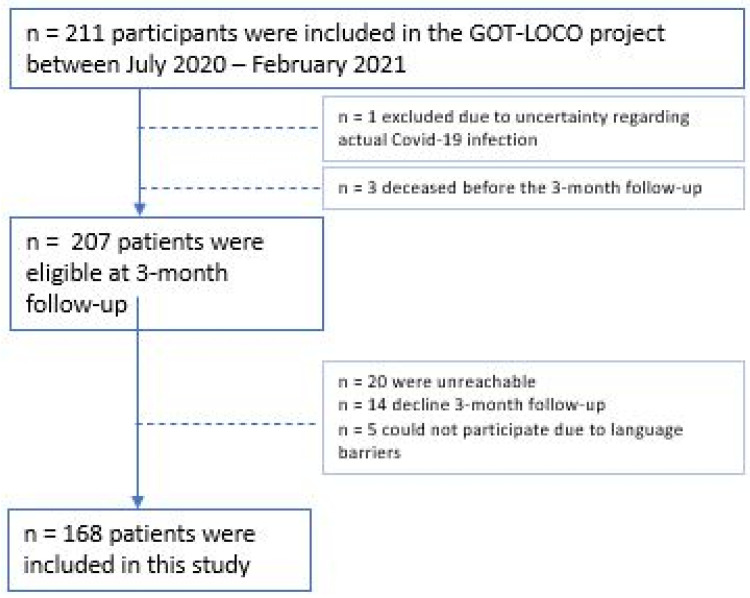
Flowchart of included participants in the GOT-LOCO.

All 211 participants were contacted by telephone and/or mail 3 months after hospital discharge and 168 agreed to participate (Figure 2). The main reason for dropout was that patients were unreachable. One hundred and fifty participants were interviewed by phone, and eighteen returned the PROMs without interview.

We found no significant differences between participants (n = 168) and non-participants (n = 39) in regard to age and sex. However, participants included in the study were treated in the ICU to a higher extent (n = 89, 53%; *p* = 0.045). Of the 168 participants, 116 (69%) were men, eighty-seven (52%) were ≥65 years old. The mean length of hospital stay was 34.6 ± 37.6 days (min 5–max 200 days) and the majority (n = 128, 77%) were discharged to their home (Table 1).

### 3.1. Body Function

Participants assessed their physical function as slightly limitated (median PCFS 2) and 103 (64%) scored ≥10 on CAT (median 14 points), indicating respiratory difficulties. The item on the CAT perceived as most difficult was “breathlessness” (48%). Regarding self-perceived traumatic stress, anxiety, and depression, the median scores were below the corresponding cut-offs (Table 2). Thirty-four participants (22%) perceived symptoms of depression and 58 (37%) symptoms of anxiety. Fifty-three (34%) had self-assessed traumatic stress due to COVID-19. Furthermore, 113 participants (72%) perceived general fatigue (≥11 on MFI-20), where general fatigue and physical fatigue were the most prominent domains (both median 15 on MFI-20). 

### 3.2. Activity

**Figure 3:**
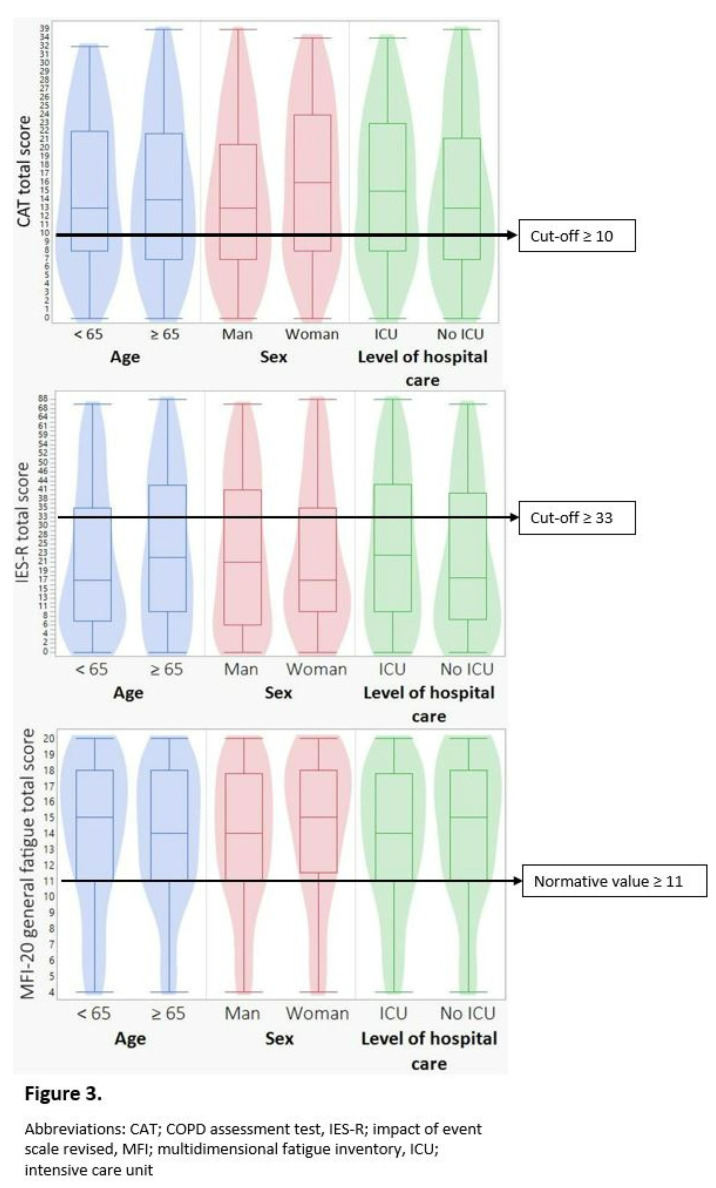
Results on CAT, IES-R and MFI-20 depending on age, sex and level of hospital care.

Half of the participants (50%) assessed their physical activity level as light (median 2 on SG-PALS). Participants treated in the ICU assessed a significantly higher level of physical activity at 3 months compared to the patients not treated in the ICU (*p* = 0.026). Furthermore, men assessed higher level of physical activity (*p* = 0.014) compared with women. Twenty-five (50%) of the women and 32 men (28%) were physically inactive (SG-PALS 1). No difference was found in perceived level of physical activity depending on age (Table 2). The majority (68%) of participants perceived their walking ability as independent (median 5 on FAC). Older participants had significantly lower walking ability (*p* = 0.001) than younger participants. No difference in walking ability was found depending on sex or level of hospital care (Table 2). Women had lower functional status (PCFS) compared to men (*p* = 0.031), but no significant differences were found between groups (age, sex, or previous level of hospital care) in other aspects of body function (Table 2, Figure 3). 

### 3.3. Participation 

Many participants reported gaps in activities between what they wanted to do but did not participate in, including leisure activities (73%), social activities (72%), and I-ADL (45%), Table 3. The most common gap in the I-ADL was “performing heavy maintenance”, with 21.3% of participants not being able to participate in that activity. Overall, women had more gaps compared with men (*p* = 0.028). No differences were seen deepening on age or level of hospital care, Table 2.

## 4. Discussion

We found that self-assessed aspects of health are still affected 3 months after hospitalization due to COVID-19, in body functions as well as in activities and participation. With few exceptions, similarly impaired aspects of health were seen across ages, sex, and levels of hospital care.

The majority of the study population had respiratory difficulties, with no differences depending on sex, age, and level of hospital care. The respiratory complications in this cohort were worse than previously reported in COVID-19 patients 1 month after hospital discharge [31]. This could be due to participants in this study to a larger extent received treatment in the ICU and had longer length of hospital stay. Nearly half of the participants in the present study, three months after hospital discharge, still experienced breathlessness when climbing stairs, indicating a possible need for spirometry screening. Though breathlessness is a common symptom of COVID-19 [1], progress of the respiratory impairments over time is not yet known. These results may indicate a need for respiratory follow-up after hospital discharge in these patients, also including respiratory physical therapy with inspiratory muscle training to manage breathlessness and regain inspiratory muscle function [32].

A substantial part of the study population (34%) scored above the cut-off of 33, indicating possible post-traumatic stress disorder (PTSD). Although the median score was well below the cut-off for possible post-traumatic stress syndrome [22], participants may still have a substantial amount of traumatic stress. Self-assessed was higher in the present study than previously reported for ICU-treated COVID-19 patients [33]. Taken together with the 37% of participants perceiving anxiety, these results highlight the necessity of awareness and assessments of aspects of mental health at follow-up.

The scores for general fatigue in the present study were considerably higher than normative data from the Swedish population [26]. It is important to consider that, even though individuals may perceive the same overall fatigue, their lived experiences may differ [23] and that the perception of fatigue relates closely to self assessed health [26]. In the present study no significant difference in perceived fatigue between men and women was seen. This finding differs from the normative data in which women perceived higher fatigue than men on all subscales [26]. In the present study, a larger proportion of the men were treated in the ICU compared to women (59% of men and 42% of women) and may, therefore, be more fatigued than usual, possibly making the groups of women and men equally fatigued. Also, it is important to consider the long hospital stays in this cohort, which could entail deconditioning and fatigue. 

The participants assessed their physical activity as light, being active for at least four hours a week. Physically active people have previously been shown to be less fatigued than a sedentary population [26]. Differences based on sex and level of hospital care were present, which supports previous findings [26]. Being critically ill from COVID-19, with a need for care in the ICU, may possibly result in higher motivation for regaining health and physical fitness. Our findings of a higher physical activity level in participants previously treated in the ICU might also perhaps be related to those patients possibly receiving more rehabilitation compared to the others, given their more severe condition and plausible greater need for rehabilitation, although these circumstances are unknown to us. It is important that all patients treated for COVID-19 are followed over time and, if needed, offered rehabilitation, regardless of their previous level of hospital care. Rehabilitation resources have been insufficient during the pandemic, making it necessary to prioritize [34], and it has been argued that a general need for increased post-Covid rehabilitation may last for months, if not years, after the pandemic [34]. 

Regarding participation restrictions presented in the study, it is important to consider that the COVID-19 pandemic may influence the possibility of participating in certain activities. Therefore, though gaps were common, the gaps reported in certain activities (e.g., cultural events and/or traveling) should be considered with caution. Apart from many other countries, Sweden had a somewhat different approach during the pandemic with no general lockdowns [35], but social distancing was recommended. Though it can be argued that it is normal to have some gaps in everyday life, the share of participants (45%) who had gaps in activities in daily life could be considered substantially high. The most commonly reported gap, “performing heavy maintenance”, indicated that participants have not recovered to their pre-Covid functional status 3 months after hospital discharge. 

Data from similar population at hospital discharge, demonstrated physical impairments, cognitive difficulties and activity limitations [2] most prominent in ICU-treated and older patients. However, 3 months after hospital discharge, these differences were not seen between ICU-treated patients and others, despite the use of PROMs. Younger participants and the ones not previously treated in the ICU may had a slower recovery process in some aspects of functioning after being discharged, or different-higher demands in life making the recovery process more complex. 

There were some limitations to this study. Firstly, this study was planned in the very beginning of the pandemic, when the testing for COVID-19 was still under development. In order not to exclude patients due to insufficient testing or possibly having to manage selection bias, this study was conducted without positive COVID-19 testing as an inclusion criterion, which might have influenced the inclusion of the very first participants. Secondly, at three-month follow-up there were twenty participants who were unreachable for unknown reasons. No differences were seen in dropout analysis between responders and non-responders apart from previous level of hospital care, where ICU treated participants responded to a higher extent.

## 5. Conclusions

At this 3-month follow-up, self-assessed aspects of health were affected after hospitalization due to COVID-19. Although fatigue, respiratory complications, and aspects of mental health were persistent, there were no major differences between groups based on age, sex, or level of hospital care. The recovery process after COVID-19 is multifaceted and affects body functions, as well as activity and participation. Thus, follow-up of this patient group is important regardless of age, sex, and previous level of hospital care, and needs to be individualized. Future studies with long-term follow-up and qualitative approach are needed in order to gain a better understanding of the long-term effects associated with COVID-19. 

## Figures and Tables

**Table 1:** Patient characteristics, at acute phase and at 3-month follow-up.

Acute Phase	All Patients (n = 168)	Under 65 Years n = 81 (48.2)	Over 65 Years n = 87 (51.8)	ICU Admitted n = 89 (53)	Non-ICU n = 79 (47)	Man n = 116 (69)	Woman n = 52 (31)
Age, years	64.3 ± 12.8	53.8 ± 8.8	74.1 ± 6.7	63.3 ± 11.5	65.5 ± 14.2	64.8 ± 11	63.3 ± 16
Male	116 (69)	55 (67.9)	61 (70.1)	68 (76.4)	48 (60.8)		
BMI kg/m^2^ n 96	28.7 ± 6.6	30 ± 8	28 ± 5.2	30 ± 7.2	27.4 ± 5.6	28.8 ± 6.1	29 ± 8
Total LOS, days	34.6 ± 37.6	34.6 ± 44.4	34.6 ± 30.1	51.6 ± 43.7	15.5 ± 13	39.7 ± 42.5	23.4 ± 19.3
ICU admission	89 (53)	45 (55.6)	44 (50.6)			68 (58.6)	21 (40.4)
Discharged to n 167							
Home	128 (76.6)	73 (91.3)	55 (63.2)	68 (76.4)	60 (76.9)	91 (79.1)	37 (71.2)
Home with community nursing assistance	26 (15.6)	7 (8.8)	19 (21.8)	15 (16.9)	11 (14.1)	18 (15.7)	8 (15.4)
Short term nursing home	13 (7.7)	-	13 (14.9)	6 (6.7)	7 (9)	6 (5.2)	8 (15.4)
At 3-month follow-up							
Current living situation n = 166							
Living alone	54 (32.5)	18 (22.2)	36 (42.4)	23 (26.1)	31 (39.7)	38 (33.3)	16 (30.2)
Living with another adult	82 (49.4)	34 (42)	48 (56.5)	53 (60.2)	29 (37.2)	56 (49.1)	26 (50)
Children living at home	25 (15.1)	24 (29.6)	1 (1.2)	9 (10.2)	16 (20.5)	17 (14.9)	8 (15.4)
Living with spouse and children	5 (3)	5 (6.2)		3 (3.4)	2 (2.6)	3 (2.6)	2 (3.8)

Data are given as count = n and percentage (%), or median and standard deviation ± Abbreviations: BMI; body mass index, LOS; length of hospital stay, ICU; intensive care unit.

**Table 2:** Patient reported outcome measures at 3-month follow-up.

	All Patients n = 168	Under 65 Years n = 81 (48.2)	Over 65 Years n = 87 (51.8)	*p* Value	ICU Admitted n = 89 (53)	Non-ICU n = 79 (47)	*p* Value	Man n = 116 (69)	Woman n = 52 (31)	*p* Value
Body Function										
CAT n = 160	13.5 (14)	13 (13)	14 (15)	*p* = 0.981	15 (14)	13 (14)	*p* = 0.418	13 (13)	14 (16)	*p* = 0.197
IES-R total score n = 154	19.5 (33)	17 (27)	21 (34)	*p* = 0.465	22 (29)	17 (28)	*p* = 0.310	22 (37)	17 (25)	*p* = 0.835
Intrusion n^a^ = 157	8 (12)	8 (11)	9 (13)	*p* = 0.582	9 (13)	6 (11)	*p* = 0.233	9 (13)	7.5 (12)	*p* = 0.986
Avoidance n^a^ = 155	7 (13)	5 (12)	7 (13)	*p* = 0.239	7 (12)	6 (12)	*p* = 0.329	7 (14)	6.5 (11)	*p* = 0.791
Hyperarousal n^a^ = 157	5 (10)	5 (9)	5 (10)	*p* = 0.674	5 (11)	5 (9)	*p* = 0.396	5 (11)	3 (9)	*p* = 0.582
MFI-20 n = 157										
General fatigue	15 (7)	15 (6)	14 (7)	*p* = 0.614	14 (6)	15 (7)	*p* = 0.423	14 (7)	15 (7)	*p* = 0.320
Physical fatigue	15 (7)	15 (7)	15 (6)	*p* = 0.249	15 (6)	15 (8)	*p* = 0.386	15 (6)	15.5 (8.75)	*p* = 0.239
Mental fatigue	11 (7)	11 (8)	11 (8)	*p* = 0.735	11 (8)	12 (6)	*p* = 0.165	11 (7.25)	11 (8.75)	*p* = 0.460
Reduced motivation	9 (5)	9 (7)	9 (4)	*p* = 0.419	9 (5)	9 (5)	*p* = 0.407	9 (5)	9.5 (6.5)	*p* = 0.155
Reduced activity	14 (6)	13 (8)	14 (6)	*p* = 0.396	13 (6)	14 (8)	*p* = 0.899	13 (7)	14 (6.5)	*p* = 0.337
HAD Anxiety n = 159	5 (7)	4 (7)	5 (5)	*p* = 0.880	4 (8)	5 (6)	*p* = 0.796	5 (6)	3.5 (6)	*p* = 0.561
Depression n = 158	3 (5)	3 (5)	3 (4)	*p* = 0.883	4 (5)	2 (4)	*p* = 0.851	3 (5)	2.5 (5)	*p* = 0.529
Activity										
PCFS n = 160	2 (4)	2 (3)	2 (3)	*p* = 0.398	2 (3)	2 (3)	*p* = 0.824	2 (3)	3 (2)	***p* = 0.031**
SG-PALS n = 160	2 (1)	2 (1)	2 (1)	*p* = 0.501	2 (2)	2 (1)	***p* = 0.026**	2 (1)	2 (1)	***p* = 0.014**
FAC n = 157	5 (1)	5 (0)	5 (1)	***p* = 0.001**	5 (1)	5 (1)	*p* = 0.683	5 (1)	5 (1)	*p* = 0.100
Participation										
OGQ n = 155	5 (6)	5 (6)	5 (6)	*p* = 0.692	6 (6)	5 (6)	*p* = 0.506	5 (7)	6 (5.75)	***p* = 0.028**

Data is presented as count n and percentage (%), and median and inter quartile range (IQR). n^a^; count of accurately filled out forms resulting in a total score in the sub category but not a total score of the form. Abbreviations: ICU; intensive care unit, CAT; COPD assessment test, IES-R; impact of event scale–revised, MFI-20; multidimensional fatigue inventory −20, HAD; hospital anxiety and depression scale, PCFS; post covid functional status, SG-PALS; salting grimby physical activity level scale, FAC; functional ambulation category, OQG; Occupational gaps questionnaire. Significant *p* values are in bold.

**Table 3:** Occupational GAPS. Abbreviations: n: count, ADL: activities of daily living.

Occupational GAPS (n = 155) Activity	WTD GAPs n %
Instrumental ADL	70 (45.2)
Grocery shopping	26 (16.8)
Cooking	17 (11)
Laundry	23 (14.8)
Cleaning	21 (13.5)
Performing light maintenance	24 (15.5)
Performing heavy maintenance	33 (21.3)
Personal finance	5 (3.2)
Transporting oneself	22 (14.2)
Leisure activities	114 (73.3)
Shopping	32 (20.6)
Participating in sports	50 (32.3)
Outdoor life	42 (27.1)
Hobbies	38 (24.5)
Cultural activities	72 (46.5)
TV/video/radio	-
Reding newspapers	11 (7.1)
Reading books or periodicals	22 (14.2)
Writing (i.e., e-mail, poems, books)	18 (11.6)
Games (i.e., boardgames, lottery, crosswords)	13 (8.4)
Using the computer	9 (5.8)
Social activities	115 (72.4)
Socializing with partner and/or children	13 (8.4)
Socializing with family and/or friends	29 (18.7)
Helping/supporting others	44 (28.4)
Engaging in societies, clubs or unions	43 (27.7)
Participating in religious/spiritual activities	9 (5.8)
Visiting restaurants and bars	58 (37.4)
Traveling	95 (61.3)
Work related activities	50 (32.3)
Working	32 (20.6)
Studying	8 (5.2)
Taking care of/raising children	5 (3.2)
Performing voluntary work	19 (12.3)

## Data Availability

The dataset analyzed for this study is not available to the public, due to Swedish ethical restrictions. More in this can be found at http://etikprovningsmyndigheten.se (assessed on 23 May 2022). Permission to use data is only for what has been given ethical approval by the Swedish Ethical Review Authority. Upon reasonable request data may be available from the authors (contact Hanna C Persson; hanna.persson@neuro.gu.se).
